# Nanostructured biosensor using bioluminescence quenching technique for glucose detection

**DOI:** 10.1186/s12951-017-0294-1

**Published:** 2017-08-22

**Authors:** Longyan Chen, Longyi Chen, Michelle Dotzert, C. W. James Melling, Jin Zhang

**Affiliations:** 1 Department of Chemical and Biochemical Engineering, University of Western Ontario, 1151 Richmond St., London, ON N6A 5B9 Canada; 2 School of Kinesiology, Faculty of Health Sciences, University of Western Ontario, London, ON N6A 5B9 Canada

**Keywords:** Bioluminescence, Biosensor, Nanoparticles, Glucose

## Abstract

**Background:**

Most methods for monitoring glucose level require an external energy source which may limit their application, particularly in vivo test. Bioluminescence technique offers an alternative way to provide emission light without external energy source by using bioluminescent proteins found from firefly or marine vertebrates and invertebrates. For quick and non-invasive detection of glucose, we herein developed a nanostructured biosensor by applying the bioluminescence technique.

**Results:**

Luciferase bioluminescence protein (Rluc) is conjugated with β-cyclodextrin (β-CD). The bioluminescence intensity of Rluc can be quenched by 8 ± 3 nm gold nanoparticles (Au NPs) when Au NPs covalently bind to β-CD. In the presence of glucose, Au NPs are replaced and leave far from Rluc through a competitive reaction, which results in the restored bioluminescence intensity of Rluc. A linear relationship is observed between the restored bioluminescence intensity and the logarithmic glucose concentration in the range of 1–100 µM. In addition, the selectivity of this designed sensor has been evaluated. The performance of the senor for determination of the concentration of glucose in the blood of diabetic rats is studied for comparison with that of the concentration of glucose in aqueous.

**Conclusions:**

This study demonstrates the design of a bioluminescence sensor for quickly detecting the concentration of glucose sensitively.

**Electronic supplementary material:**

The online version of this article (doi:10.1186/s12951-017-0294-1) contains supplementary material, which is available to authorized users.

## Background

Glucose is one of the most important carbohydrates to provide energy for cell metabolism. Studies also show that glycolytic flux is closely related to enzyme levels. Control of glucose level is the key to the regulation of signaling protein-involved pathways, and to manage chronic diseases, e.g. diabetics [1]. Different techniques from electrochemical methods to fluorescence detections have been developed for monitoring glucose level in vitro and in vivo [2–4]. Biosensors by using luminescence techniques have advantages in directly converting bioprocess to luminescence signal through a non-toxic and cost-effective process [5]. Resonance energy transfer (RET) techniques including Förster/fluorescence RET (FRET) and bioluminescence RET (BRET) are distance-dependent non-radiation energy transfer, which have been used for detecting protein–protein interactions in vitro and in vivo [6–8].

Generally, both FRET and BRET utilize the resonance energy transfer from a fluorophore donor to a fluorophore acceptor. The resonance energy transfer is dependent on the distance between donor and acceptor. Recent developments on FRET biosensor unveil a quenching mechanism due to the resonance energy transfer between metallic nanoparticles (NPs) and fluorophore donor. The quenching mechanism allows luminescence biosensor to obtain high signal-to-noise ratio, in which the fluorescence intensity of the donor can be quenched by the acceptor made of metallic nanoparticles, and the fluorescence intensity of the donor can be restored if the distance between the donor and metallic NPs increases [9, 10]. Metallic NPs, e.g. gold nanoparticles (Au NPs), can be used as an acceptor to quench the fluorescence of a donor in a FRET sensor due to their large extinction coefficients and broad energy absorption in the visible range. The quenching efficiency is reverse proportional to the distance between nanoparticle and the donor in a designed FRET biosensor [11–13]. The FRET assays by using quenching mechanism have been applied in different organic-based assays for nucleic acid detection [14, 15].

Bioluminescence is a unique phenomenon in nature which can be found from firefly to marine vertebrates and invertebrates. Currently, bioluminescence techniques have been used in studying gene expression. BRET technique shows an advantage over FRET technique because it does not require the external energy, e.g. light source, to excite the donor. Therefore, BRET technique has been used for real-time and non-invasive detection [16, 17]. Normally, bioluminescence reaction happens when luciferase, an enzyme, reacts with the substrate, e.g. coelenterazine (CTZ) [18, 19]. Luciferase protein can be used as a donner in BRET biosensor [20, 21]. Quite recently, luciferase protein conjugated onto Au NP has been demonstrated for proteases detection [16]. The bioluminescence intensity of luciferase can be eliminated/quenched by the conjugated Au NPs through the resonance energy transfer process. To our best knowledge, no such system has been applied in detection of analytes other than proteases.

**Scheme 1:**
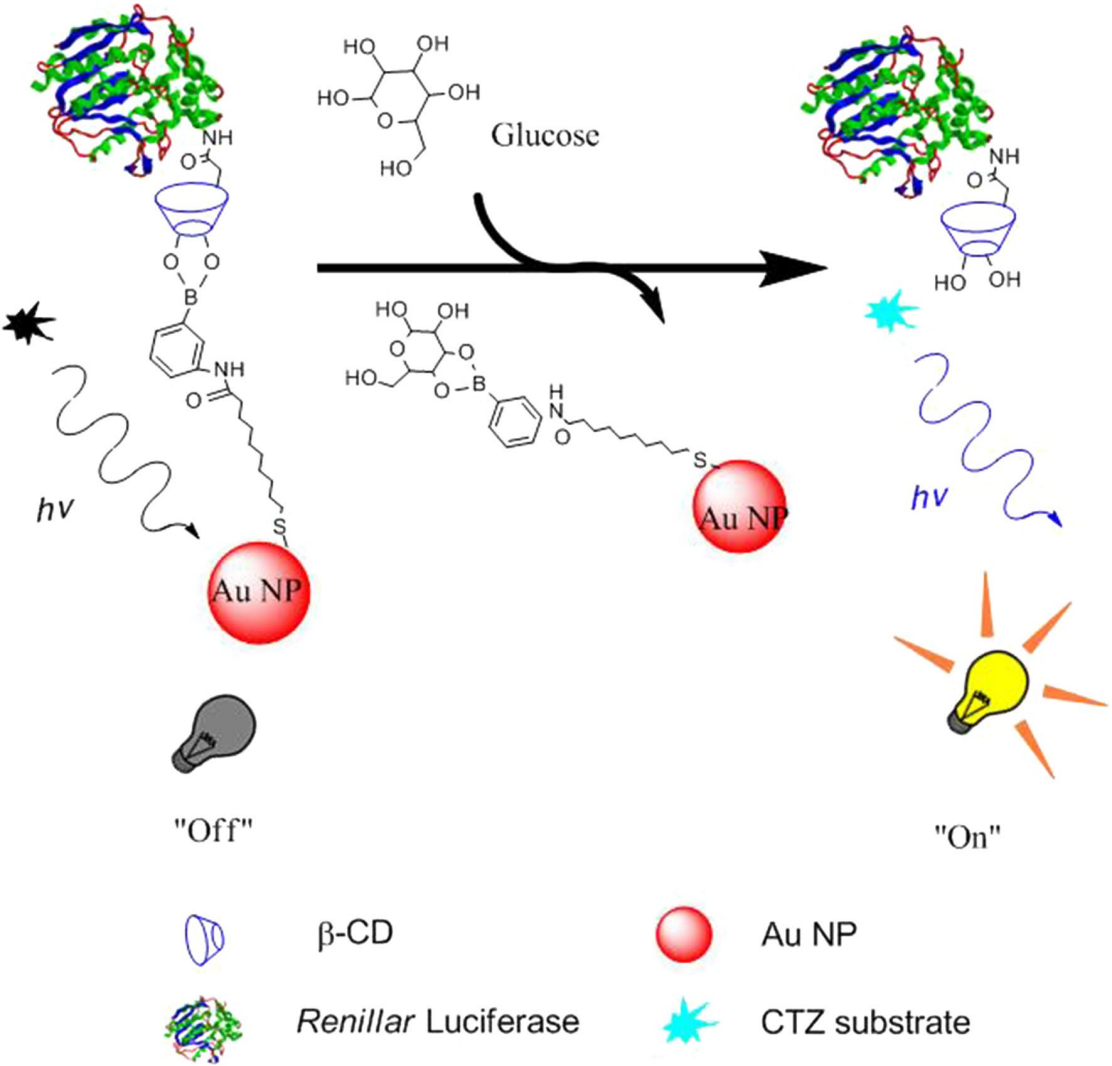
Schematic illustration of bioluminescence quenching-based nanosensor in glucose sensing

Here, we developed a bioluminescence sensor by applying bioluminescent quenching principle for detecting glucose in a homogeneous assay format. The nanostructured biosensor is composed of a donor, i.e. Renilla luciferase conjugated with β-cyclodextrin (β-CD-Rluc), and a quenching element made of Au NPs modified with phenylboronic acid (PBA) as shown in Scheme 1. It is known that PBA can react with glucose by forming cyclic ester [22, 23]. PBA has been used as a recognition element for glucose sensing [24, 25]. Once Au NPs modified with PBA react with β-CD-Rluc through a reversely covalent bond as shown in Scheme 1, Au NPs can quench the bioluminescence intensity generated by Rluc reacting with coelenterazine (CTZ). In the presence of glucose, the bioluminescence intensity will be restored due to the stronger interaction between glucose and PBA, which leads to the release of β-CD-Rlu from PBA-Au NPs. In this study, we have investigated the effect of the ratio of the donor to the quenching on the optimal bioluminescence intensity with/without glucose. The study demonstrates the restored bioluminescence intensity as a function of the concentration of glucose.

## Methods

*Renilla reniformis* luciferase gene (*Rluc*) was purchased from Promega Inc. The vector pET-32a (EMD Millipore Inc.) was used to subclone the *Rluc*. The *E. coli* strain BL21 (DE3) (invitrogen) was used as the bacterial strain for the expression of proteins. Restriction enzymes were purchase from New England Biolabs Inc. Unless otherwise indicated, all the chemicals were purchased from Sigma-Aldrich. Ultrahigh quality water with a resistance of 18.2 MΩ cm (at 25 °C) was obtained from a Nanopure™ water system (Thermofisher) fitted with a 0.22 µM filter.

### Preparation of Au NP-based acceptor of the BRET sensor

Citrate-stabilized gold nanoparticles (Au NPs) were synthesized by using the reported method [26]. In brief, 240 mL of aqueous solution containing 0.21 mM of HAuCl_4_ (0.08 mg/mL) and trisodium citrate (13.8 mg) was mixed at room temperature. The mixture was added 5 mL of ice-cold 0.1 M NaBH_4_ solution under vigorous stirring. The solution was aged overnight in a dark place with vigorous stirring. Citrate-stabilized Au NPs were further modified by 11-mercaptoundecanoic acid (MUA). 20 mL of citrate-stabilized Au NPs (60 nM, pH pre-adjusted to 10.3 by 2 M NaOH) was mixed with 19 mL of ethanol. 1 mL of MUA (10 mM) ethanol solution was added in the gold solution and stirred for 19 h (hrs). The mixture was then filtered through 0.45 μm acetate filter and purified by Amicon ultralfilter (100 kDa MWCO). Finally, the MUA modified particles were re-suspended in 20 mL water (~60 nM).

MUA-modifying Au NPs (MUA-Au NPs) was further conjugated with 3-aminophenylboronic acid (PBA) through carbodiimide reaction. Briefly, MUA-Au NPs (60 nM) was mixed with PBA (10 mg/mL), 1-ethyl-3-(3-dimethylaminopropyl)-carbodiimide (EDC, 10 mg/mL), and *n*-hydroxysuccinimide (NHS, 10 mg/mL) for 2 h at room temperature with gently shaking. The conjugated particles were further purified by Amicon ultrafilter (MWCO 100 kDa) and resuspended in phosphate-buffered saline buffer (PBS, 10 mM, pH 7.4).

### Preparation of Rluc protein

The entire coding sequences of *Rluc* was cloned to the MCS site of pET 32-a plasmid under two restriction sites (BamH I and Xho I). Two primers were designed for the cloning (forward: 5′AAAGGATCCAGCGGTGGTGGTGGTAGCATGACTTCGAAAGTTTATGATCCAG; reverse: 5′ TGTGCTCGAGTTGTTCATTTTTGAGAACTCGCTC 3′). A trx region from pET 32-a coding for thioredoxin protein is kept to maintain high level of recombinant protein expression [27]. A six-amino acid linker (SGGGGS) highlighted by the underline in the forward primer was inserted after BamH I site to leave a flexible space for proper folding of Rluc protein. The successful construction of the plasmid was confirmed by DNA sequencing (Robarts Institute, Western University, London, ON).

For protein expression, the bacterial cells (*E. coli* k-12) with recombinant plasmid were grown from a single colony overnight at 37 °C in 5 mL of Luria–Bertani (LB) broth (containing 100 μg/mL ampicillin). After transferring to 500 mL of LB broth, the cells were allowed growing for 2 h at 37 °C. The cells were then cultured with isopropyl β-D-1-thiogalactopyranoside (IPTG) at a final concentration of 1 mM for 4 h at 20 °C. The cells were harvested by centrifugation at 12,000 rpm for 5 min at 4 °C, and the pellet was resuspended in a lysis buffer [20 mM Tris/HCl buffer (pH 7.4), containing 500 mM NaCl, 5 mM imidazole, 0.2 mg/mL of lysozyme and 0.1% Triton-X-100] and sonicated with ice cooling for 15 s (s) followed by 30-s rest for 20 cycles by a Mandel Scientific Q500 sonicator. The suspension was centrifuged at 10,000 rpm at 4 °C for 30 min. The supernatant containing overexpression protein was purified via His-trap HP columns (GE lifescience, Inc.) by a syringe pump. After loading the samples, the expressed protein was eluted with an elution buffer [200 mM imidazole, 20 mM Tris/HCl buffer (pH 7.4), 500 mM NaCl and 10% glycerol] and dialyzed with PBS using Pur-A-Lyzer™ Mega Dialysis Kit (12KDa MWCO, Sigma-Aldrich). The purified protein was further concentrated by an Amicon Ultra centrifugal filter (ultra-15, MWCO 10 kDa, EMD Millipore Inc). The resultant Rluc protein solution was stored in aliquot at −80 °C. The concentration of the protein was determined by bicinchoninic acid (BCA) protein assay (Thermo scientific Inc).

### Conjugation of β-cyclodextrin (β-CD) to Rluc

Dimethylformamide (DMF) solution containing 3.78 mg of succinyl-β-cyclodextrin (~2 µmol) was mixed with 250 µL of 10 mg/mL NHS and 400 µL of 16 mg/mL EDC. The mixture in 350 µL PBS was incubated for 2 h at room temperature under gently shaking. For conjugating reaction, 200 µL of above solution was mixed with 200 µL of 10 mg/mL Rluc solution in a PBS solution (final volume was maintained at 1 mL). The solution was further incubated overnight at 4 °C. The reaction was terminated by addition of 5 µL of ethanolamine. The β-CD labeled Rluc (β-CD-Rluc) was purified through a Nap-10 column (GE Healthcare) with PBS as an eluent. The labeled protein was collected by Amicon ultral filter (ultra-15) to desired concentration and stored at 4 °C for at least 4 weeks which can maintain over 90% activity. For long-term storage, the labeled protein was stored at −80 °C with complete retention of activity.

### Carbohydrate assay

The amount of β-CD modified on a Rluc was determined by a sulfuric acid-phenol assay with slight modification [18]. The detailed results can be found from the supporting documents. In a typical test, a 30 μL of ice-colded glucose standard samples or β-CD-Rluc solution was mixed with 100 μL of concentrated sulfuric acid in microplate wells. 20 μL of aqueous 5% phenol solution was then added into the well. The plate was floated uncovered on near boiling (>90 °C) water bath for 5 min for color development, followed by cooling on ice for another 5 min. A microplate reader (Tecan M200 multimode microplate reader) was used to collect the absorbance of each well at 490 nm. The concentration of standard solution was plotted with the absorbance of each solution. The molar concentration of β-CD was thus calculated. The number of β-CD per protein was obtained by dividing the molar concentration of β-CD over the molar concentration of protein Rluc.

### Evaluation of the bioluminescence quenching-based assay

3 µL of β-CD-Rluc (1 µM) was mixed with 100 µL of PBA-Au NPs (30 nM) in a PBS solution containing 0.1% bovine serum albumin (BSA). BSA was added to stabilize Rluc activity and reduce non-specific binding between protein and Au NPs. 10 µL of glucose solution (with different concentrations) was added in the above solution. For the reactivity assay, 10 µL of all the tested substances with concentration of 50 µM was added into the above solution. The final incubation volume was 300 µL. The solution was then incubated at room temperature for 30 min. The bioluminescence was then collected by adding 1 µL of native coelenterazine (CTZ, 1 mg/mL in ethanol) in the above solution.

Male Sprague–Dawley rats, 8-weeks of age, were obtained from Charles River Laboratories. Rats were housed two per cage at constant temperature and humidity on a 12 h dark/light cycle, and had access to water and standard chow ad libitum. Ethics approval was obtained from the University of Western Ontario Research Ethics Board, in accordance with Canadian Council on Animal Care guidelines. The experimental protocol followed the Principles of Laboratory Animal Care (US NH publication No83-85, revised 1985). Diabetes was induced with multiple low-dose Streptozotocin (STZ) injections. STZ (20 mg/kg; Sigma Aldrich, Oakville, ON, Canada) in citrate buffer was injected into the intraperitoneal cavity for 5 consecutive days, and the blood samples of diabetic rats were measured by glucometer to verify the glucose concentration is larger than 20 mM. Diluted blood samples of diabetic rats with varied glucose concentrations, i.e. 100, 50, 20, 10, 5 and 1 μM, were prepared for evaluating the performance of the nanostructured sensor.

### Materials characterization

Au nanoparticles with different surface modification were characterized by transmission electron microscope (TEM), UV–Vis spectrometer, and Fourier transform infrared spectroscopy (FTIR).

Transmission electron microscopy (TEM) image was obtained through a Philips CM-10 operating at 100 kV. Dynamic light scattering (DLS) measurements were performed on a Zetasizer Nano-ZS (Malvern Instruments Inc.) equipped with a He/Ne laser at 633 nm. UV–Vis spectroscopic measurements were obtained on a Cary 300 spectrometer. Fourier transform infrared (FT-IR) spectra were conducted within the 4000–500 cm^−1^ wavenumber range using a Bruker FTIR-IFS 55 spectrometry. Bioluminescence measurements were recorded by using a QuantaMaster 40 Spectrofluorometer (Photon Technology International Inc., London, ON). The software was set up at emission collection. The shutter at laser path was closed and the emission spectra were collected from 430 to 580 nm in a mode of 2 nm per step and integration time at 0.2 s.

## Results

**Fig. 1:**
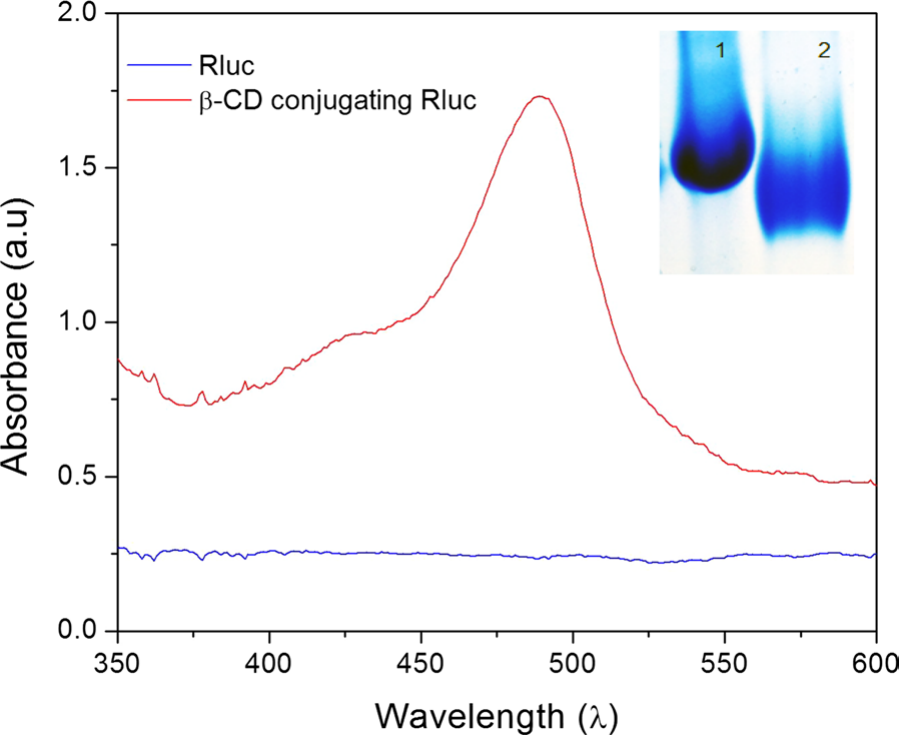
UV–vis spectra of the Rluc with/without the conjugation of β-CD. The small inset image is the native-PAGE characterization, where *Lane 1* is Rluc without conjugation, and *Lane 2* refers β-CD conjugating-Rluc

**Fig. 2:**
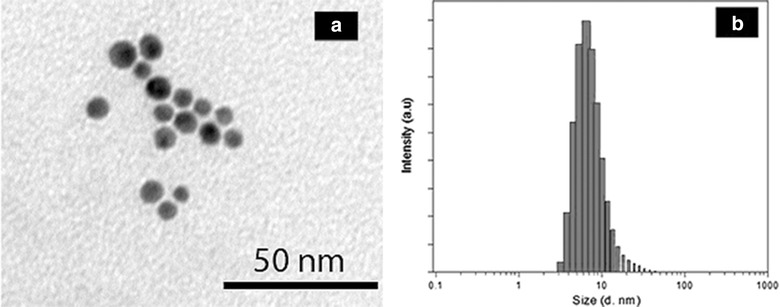
**a** TEM micrograph of citrate acid capped Au NPs; and **b** DLS analysis of the citrate acid capped Au NPs

**Fig. 3:**
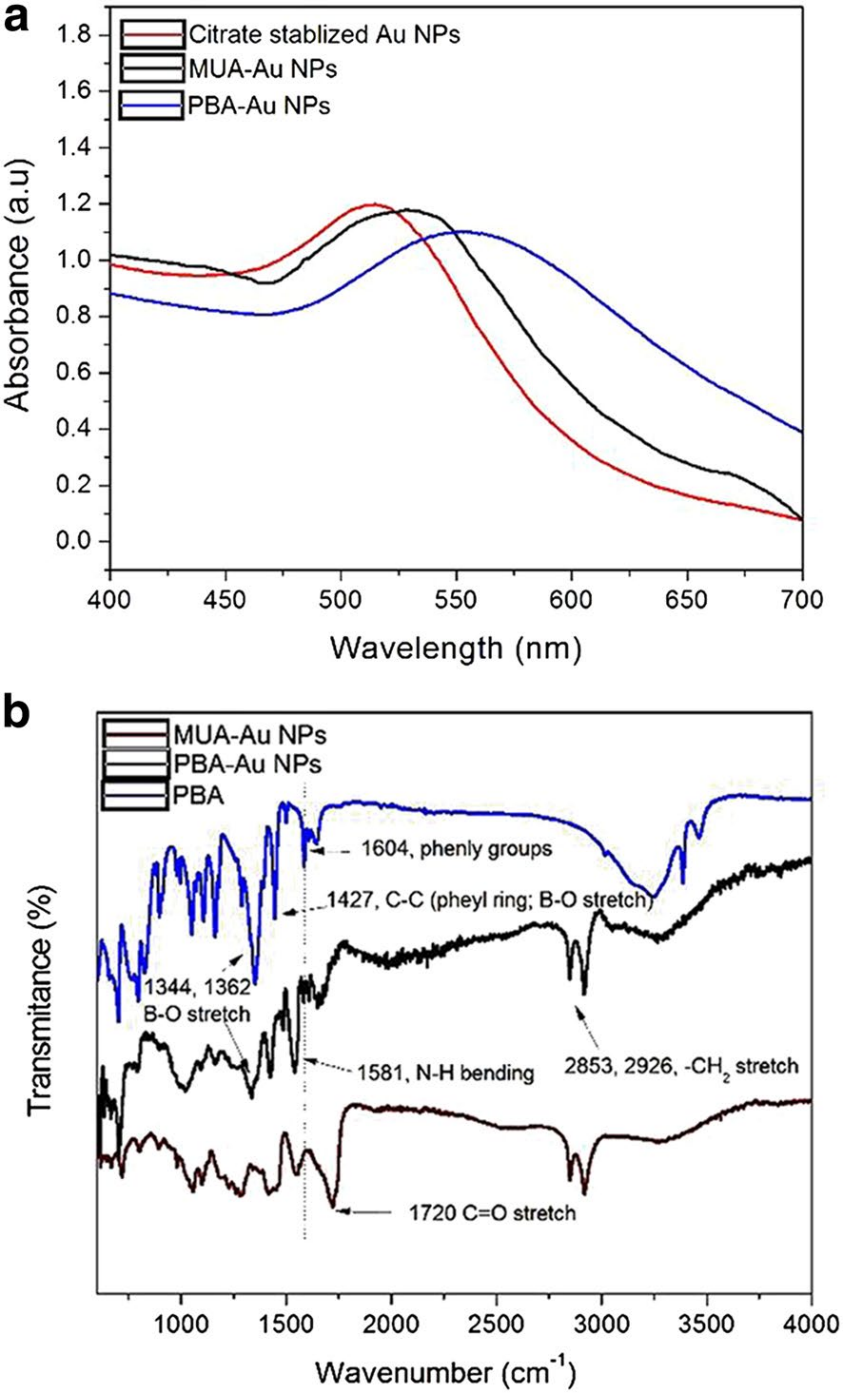
Characterization of the acceptor of the BRET sensor. **a** UV–vis spectra of Au nanoparticles with surface modifications. **b** FTIR spectra of Au NPs with surface modifications

Carboxyl-modified β-CD was conjugated onto Rluc protein through the carbodiimide-mediated reaction, Native polyacrylamide gel electrophoresis (native-PAGE) was used to investigate the conjugation of carboxyl-modified β-CD to Rluc. It is noted that the β-CD conjugation could decrease protein positive charges because of the formation of primary amine group. Hence, the proteins with or without the conjugation of β-CD could be separated by native-PAGE on basis of protein surface charges [17]. In Fig. 1, the sample of β-CD-Rluc (lane 2) displays significant band shift as compared to the negative control, i.e. Rluc without conjugation (lane 1). Meanwhile, the conjugation of β-CD onto Rluc protein was evaluated by using sulfuric acid-phenol colorimetric assay for total carbohydrate after purification [18, 19]. In Additional file 1: Figure S1, the typical peak at 490 nm to the sample of β-CD conjugating Rluc is observed, which confirms the successful conjugation of β-CD to the protein. The optimized molar ratio of β-CD to Rluc protein is estimated at 100:1, that is, 10 β-CD molecules on each Rluc molecule, which is determined through the standard curve (Additional file 1: Figure S1, ESI†). The precipitation of protein is observed when the molar ratio of β-CD to Rluc is larger than 100:1, which might be related to the high co-solvent (dimethylformamide) concentration in buffer [28]. TEM and DLS were carried out to measure the average particle size and size distribution of Au NPs. Figure 2a is the TEM micrograph of Au NPs. The spherical NPs have the average particle size around 8 ± 3 nm. While, the result of DLS indicates that average particle size is around 10 nm with large size distribution as shown in Fig. 2b. In addition, Fig. 3a shows the UV–Vis spectra of Au NPs with different modification. The citrate-stabilized Au NPs have typical surface plasmon resonance peak at 512 nm. The localized surface plasmon resonance (LSPR) of Au NP with the modification of MUA and PBA is centering at 525 and 550 nm, respectively. The broaden absorption band could be related to the changes in the local environment and chemical interface dampening [29]. The surface modifications on Au NPs was further investigated by FTIR spectra. In Fig. 3b, the peaks around 1344 cm^−1^ (to 1362 cm^−1^) are attributed to B-O stretch from PBA [30]. Phenyl ring signature bands are observed at 1427 cm^−1^ (strong) and 1604 cm^−1^ (weak) to PBA-Au NPs [22]. The N–H bending band (red dash circle in Fig. 2b) is shown at 1581 cm^−1^, which appears relative weak peak to the sample of PBA-Au NPs [25]. The amide I band of PBA-Au NPs associated with –C=O stretch of peptide bond spans from 1600 to 1700 cm^−1^ [31]. The results of FTIR indicates that successful modification of Au NPs with PBA.

**Fig. 4:**
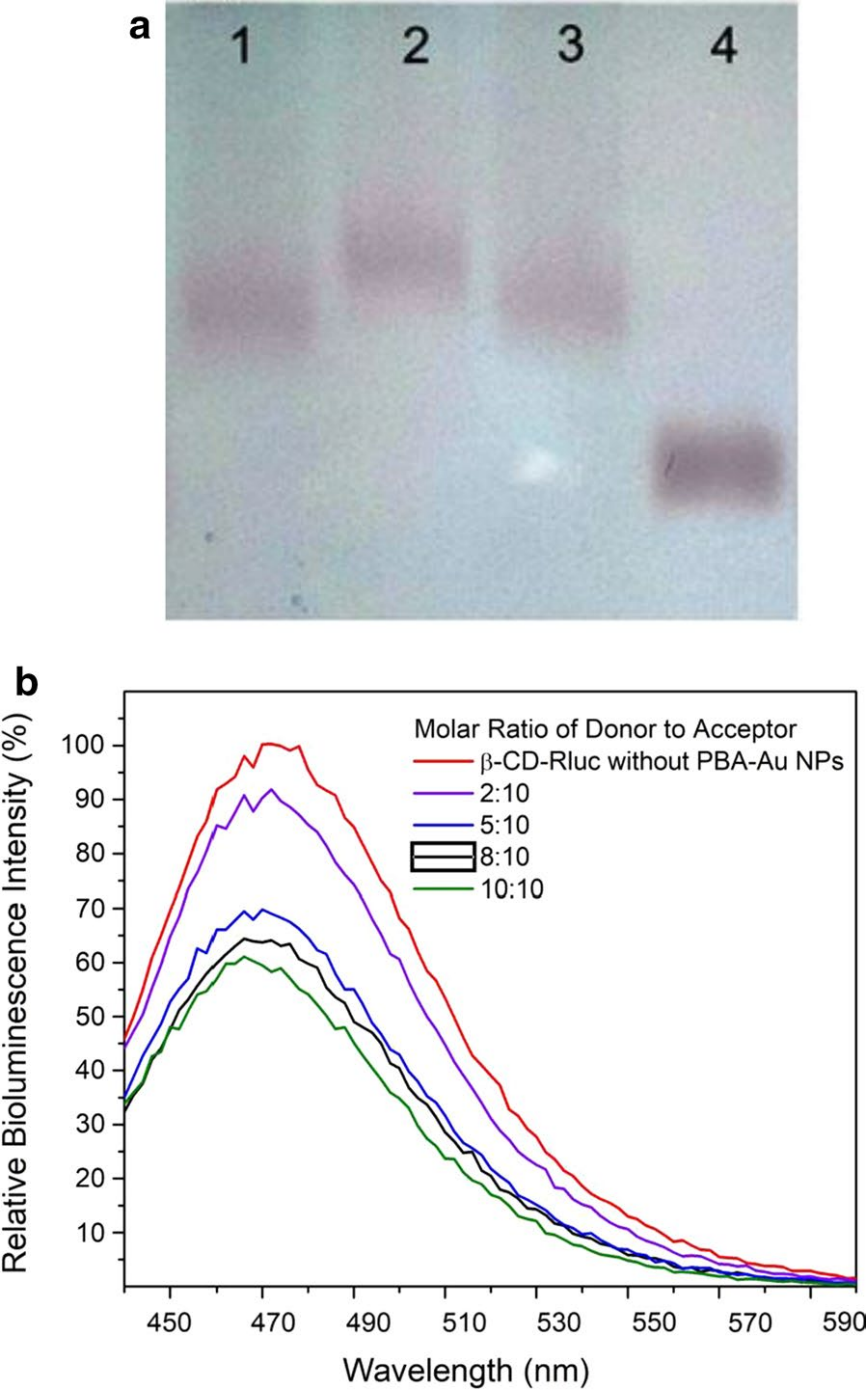
Analysis of the interaction between PBA-Au NPs and β-CD-Rluc. **a** Agarose gel electrophoresis (0.5%); the concentration of β-CD-Rluc is 10 µM. In agarose gel electrophoresis, the samples from *left to right* are, PBA-Au NPs alone (*lane 1*), PBA-Au NPs reacted with β-CD-Rluc (*lane 2*), PBA-Au NPs incubated with Rluc (unmodified Rluc, *lane 3*) and MUA-Au NPs (*lane 4*), respectively. **b** Bioluminescence spectra of β-CD-Rluc responding to the addition of PBA-Au NPs with different molar ratio

PBA-Au NPs then react with β-CD-Rluc through the reversible boronic acid-diol interaction [22]. The interaction between PBA-Au NPs and β-CD-Rluc was investigated by agarose gel electrophoresis. In Fig. 4a, the mixture of PBA-Au NPs and β-CD-Rluc displays the slowest migrating band (lane 2) as compared to the negative controls, i.e. PBA-Au NPs alone (lane 1), PBA-Au NPs mixing with unmodified Rluc (lane 3), and MUA-Au NPs alone (lane 4). This band shift could be attributed to an increase of NPs in terms of particle size, due to the formation of β-CD-Rluc/PBA-Au NP complex. Without β-CD conjugation, Rluc/PBA-Au NP mixture shows similar band position to PBA-Au NP. Thereby, the interaction of conjugated protein with PBA-Au NP is specific due to the boronic acid-diol interaction. The bioluminescence of Rluc is quenched by the conjugated Au NPs. Figure 4b shows that the bioluminescence intensity decreases gradually when adding more PBA-Au NPs in β-CD-Rluc by comparing to the signal from control group without NPs (red curve). The bioluminescence intensity can be quenched over ~58% when the molar ratio of PBA-Au NPs to β-CD-Rluc in the mixture is increased to 10/10 as shown in Fig. 4b.

**Fig. 5:**
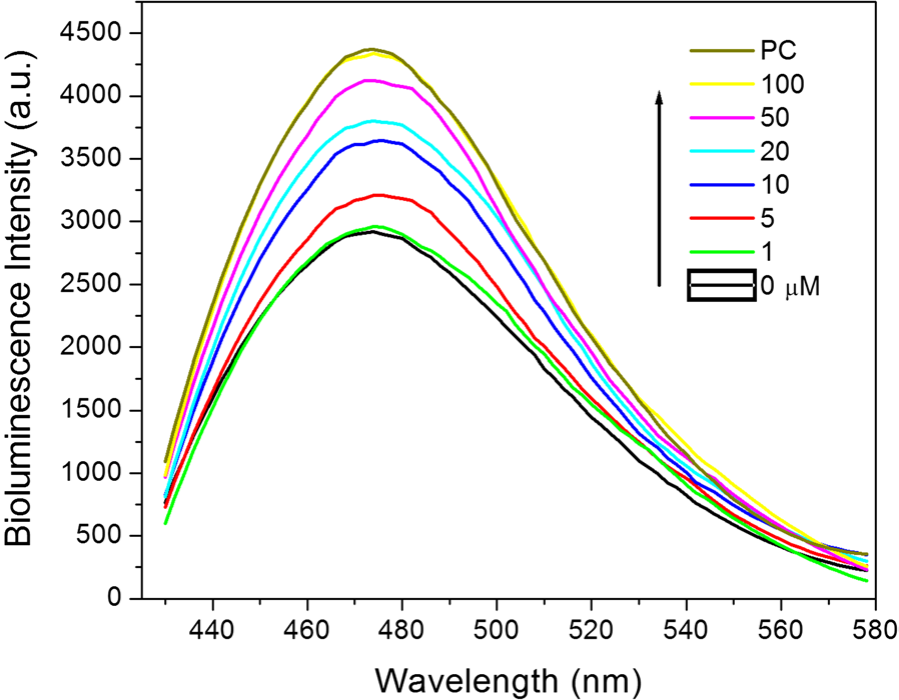
Bioluminescence intensity with different glucose concentrations. PC refers the positive control, i.e. β-CD-Rluc alone without PBA-Au NPs

Glucose intends to compete with β-CD-Rluc to react with PBA-Au NPs because the interaction between PBA and glucose is stronger than the reaction of β-CD with PBA, which, therefore, results in the restore of bioluminescence as shown in Scheme 1. The bioluminescence intensity of β-CD-Rluc as a function of the concentration of glucose was further investigated. In a typical assay, the nanostructured sensors (the molar ratio of NPs to protein = 10:10)were mixed with glucose at different concentrations, respectively.. The mixtures were incubated for 30 min. Figure 5 shows the bioluminescence intensity increases when the concentration of glucose in aqueous increases from 0 to 100 µM. It is noted that the positive control (PC) refers the β-CD-Rluc which has the same amount of β-CD-Rluc in the designed sensor.

The normalized bioluminescence intensity was calculated by using the Eq. 1;

1 $${\text{Normalized Bioluminescence Intensity }} = \frac{\text{Bioluminescence Intensity in the presence of glucose }}{\text{Bioluminescence Intensity of the positive control}}$$

**Fig. 6:**
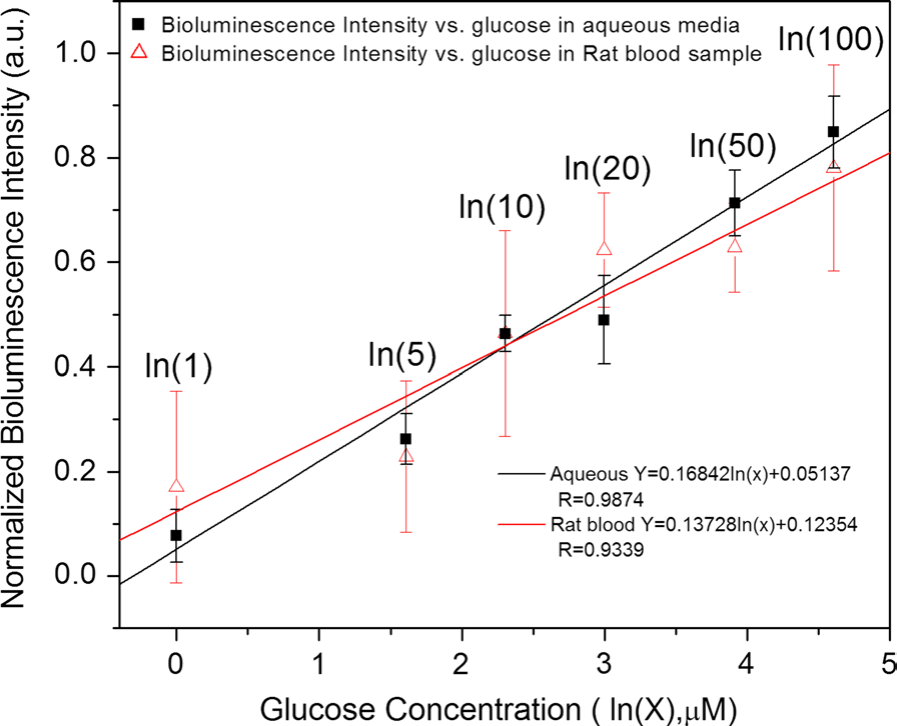
Correlation between normalized bioluminescence intensity and the logarithmic glucose concentration in aqueous and in blood samples

In addition, we tested the sensor performance with blood sample of diabetic rats to evaluate the interference of other proteins in blood. Figure 6 indicates the correlation between the normalized bioluminescence intensity and the log form of the concentration of glucose in aqueous samples and in diluted blood samples. The normalized bioluminescence intensity increases linearly when the logarithmic glucose concentration increases in a range from 1 to 100 µM to both measurements in aqueous and in diluted blood samples as shown in Fig. 6. The detection limit for this assay is secured to 1 µM from three independent measurements. Meanwhile, the normalized bioluminescence intensity corresponding to the log form of glucose in blood samples is very close to the log form of glucose in aqueous sample. Consequently, this bioluminescence nanosensor can detect plasma glucose for diabetic rats.

## Discussions

Bioluminescence reaction happens in nature when the enzyme, Rluc, reacts with a substrate, CTZ. We used the typical biological process to obtain pure and high amount of Rluc used as the donor in our designed BRET biosensor; first a plasmid containing Rluc gene was constructed, then the Rluc gene was cloned and expressed in bacterial system. High concentrated Rluc protein was obtained after the protein purification, and was conjugated to β-CD as shown in Fig. 1. Meanwhile, the acceptor was made by modified Au NPs which is able to quench the bioluminescence through the BRET mechanism. The particle size and size distribution were characterized by TEM and DLS as shown in Fig. 2. The discrepancy of the results in size distribution may result from the surface modification of Au NPs by PBS which cannot be shown up on the TEM micrographs as organic molecules have very low electron density. The surface modification of Au NPs were studied and verified by FTIR as shown in Fig. 3. In the presence of glucose, the reaction of glucose with PBA competes against the reaction of β-CD with PBA, which leads to the release of Au NPs from Rluc and results in the restoration of the bioluminescence of Rluc as shown in Fig. 4. The maximum number of β-CD conjugated onto one Rluc is estimated at 10 as shown in the supplementary materials. Figure 4b indicates that the bioluminescence intensity of Rluc can be quenched over ~58% when the molar ratio of PBA-Au NPs to β-CD-Rluc in the mixture is increased to 10:10. The restoration of bioluminescence has a linear relationship with the logarithmic concentration of glucose in a range from 1 to 100 µM as shown in Fig. 5.

To further study the sensitivity and selectivity of the designed sensor, the glucose-specific detection was evaluated by investigating the response of restored bioluminescence intensity to different molecules. The results indicate that the bioluminescent sensor does not react with amino acids, lipids, and common sugars, i.e. mannose, lactose and maltose as shown in Additional file 1: Figure S2. However, sucrose and fructose exhibited higher reactivity to the nanostructured biosensor than glucose. This could be due to higher affinity constants of fructose to PBA than glucose [32, 33]. Meantime, as sucrose is a disaccharide composed of glucose and fructose units linked via their anomeric carbons, it could be expected that one or both monosaccharides could react with PBA-Au NPs.

Though high concentration of fructose may interfere the glucose detection by using the bioluminescent sensor, it should be noted that the concentration of fructose in human blood (both healthy and diabetes subjects) is very low, almost one thousand-fold lower than glucose concentration in healthy subject [34]. Meantime, sucrose is not commonly present in human blood in healthy subjects, as it is rapidly absorbed and hydrolyzed into its monosaccharide units in the small intestine [35, 36]. In addition, Fig. 6 indicates that the results of the blood measurement for diabetic rats is very close to the results of the glucose measurement in aqueous. It is noted that the serum levels of albumin, triglycerides, total protein and glucose in rats is compatible with human’s. STZ induced diabetic rats have been well accepted as an animal model for diagnosis and treatment of diabetes [37, 38]. Consequently, this nanostructured sensor by applying the bioluminescence quenching technique can be used to detect glucose with a high sensitive and high selective fashion.

## Conclusions

In conclusion, we have developed a new nanostructured biosensor by applying bioluminescence quenching technique for detecting glucose. Bioluminescent protein, Rluc, covalently conjugated with β-CD, is used as the donor in the designed biosensor, while PBA-Au NPs act as the acceptor. The PBA-Au NPs interacts β-CD-Rluc via a reversible covalent bonding results in the quenched bioluminescence of Rluc. In the presence of glucose, it competes with β-CD to react with PBA, which releases Au NPs away from β-CD-Rluc, and results in the restoration of bioluminescence of Rluc. This paper also demonstrates the effect of the molar ratio of donor and acceptor on the bioluminescence quenching efficiency. The results indicate that normalized bioluminescence intensity increase linearly with increasing logarithmic glucose concentration in a range from 1 to 100 µM, with detection limit of 1 µM. This nanostructured bioluminescence quenching-based sensor could be feasible for blood glucose level monitoring with sample pre-treatment (e.g. dilution).

## Additional file

**Additional file 1: Figure S1.** (a) Standard curve for sulfuric acid-phenol carbohydrate assay. (b) Calculated number of β-CD conjugated on Rluc after purification verse different molar ratios in the initial carbodiimide reactions. **Figure S2.** Response of bioluminescent nanosensors to common biological substances.
