# Supplementary material for: Nanostructured biosensor using bioluminescence quenching technique for glucose detection
Source: J Nanobiotechnology. 2017 Aug 22;15:59. doi: 10.1186/s12951-017-0294-1 (PMC5567885; doi:10.1186/s12951-017-0294-1)
Supplement: Supplementary file 1 — Additional file 1: Figure S1. (a) Standard curve for sulfuric acid-phenol carbohydrate assay. (b) Calculated number of β-CD conjugated on Rluc after purification verse different molar ratios in the initial carbodiimide reactions. Figure S2. Response of bioluminescent nanosensors to common biological substances. [file 12951_2017_294_MOESM1_ESM.docx]

**Supplementary Materials**

**Nanostructured Biosensor Using Bioluminescence Quenching technique for Non-invasive Detection of Glucose**

Longyan Chen^1^, Longyi Chen^1^, Michelle Dotzert^2^, Jamie Melling ^2^, Jin Zhang^1^*

^1^ Department of Chemical and Biochemical Engineering, Western University, London, Ontario, Canada, N6A 5B9

^2^ School of Kinesiology, Faculty of Health Sciences, University of Western Ontario, London, Ontario, Canada, N6A 5B9

**S1. Quantitative analysis of the designed donor**


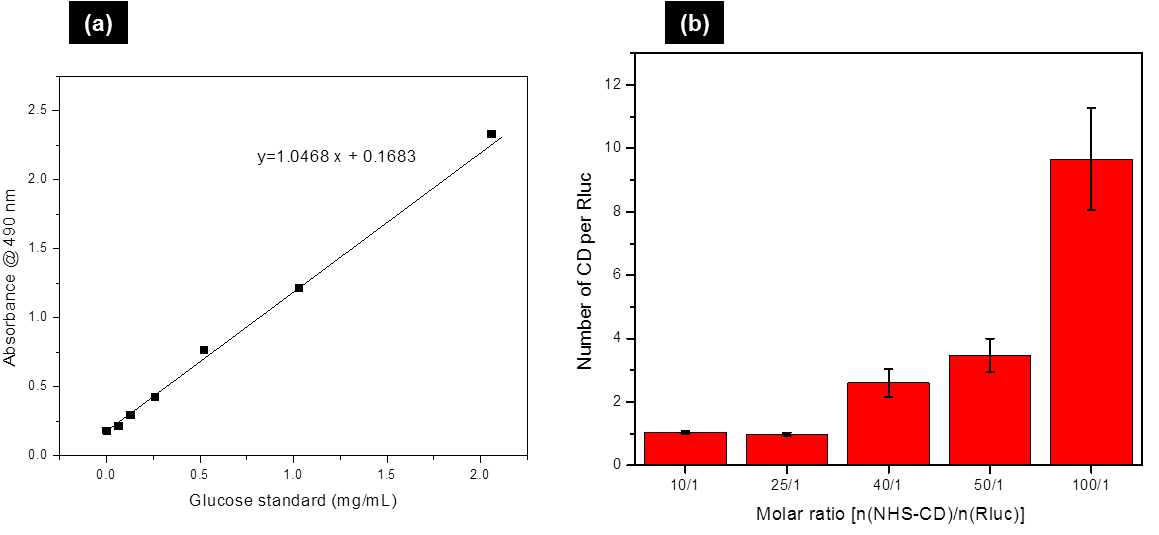


Figure S1. (a) Standard curve for sulfuric acid-phenol carbohydrate assay. (b) Calculated number of β-CD conjugated on Rluc after purification verse different molar ratios in the initial carbodiimide reactions.

The protein concentration was maintained to 30 μM in the reaction. The maximum number of β-CD conjugated onto one Rluc is estimated at 10; the molar ratio of NHS-β-CD to Rluc is 100:1.

**S2. Evaluation of the specificity of the bioluminescent nanosensor**





Figure S2. Response of bioluminescent nanosensors to common biological substances. The concentrations of all the substances are 50 µM.

Here the normalized bioluminescence is calculated by using the equation below;

Normalized bioluminescence = Relative bioluminescence intensity after adding a certain substance-relative bioluminescent intensity without any substance
